# A WEEV 6K viroporin activates MLKL-dependent necroptosis to drive inflammatory injury

**DOI:** 10.1038/s41420-026-03229-1

**Published:** 2026-07-02

**Authors:** Ziyan Zhou, Pengpeng Wu, Zhaobing Gao, Bingqing Xia

**Affiliations:** 1 State Key Laboratory of Drug Research, Shanghai Institute of Materia Medica, Chinese Academy of Sciences, Shanghai, China; 2 University of Chinese Academy of Sciences, Beijing, China

**Keywords:** Necroptosis, Drug development

## Abstract

Neurotropic alphavirus infection is characterized by neuronal injury and sustained neuroinflammation, yet the viral determinants linking membrane perturbation to host inflammatory damage remain poorly defined. Here, we show that the Western equine encephalitis virus (WEEV) 6K protein functions as an ER-localized viroporin, displaying stable single-channel activity with broad cation permeability. Consistent with its ion channel activity, 6K expression perturbs intracellular Ca²⁺ store homeostasis and is associated with cellular injury phenotypes. Mechanistically, 6K predominantly engages MLKL-mediated necroptosis, identifying programmed necrotic signaling as a principal pathway underlying 6K-driven injury under these conditions. Importantly, genetic deletion or pharmacological inhibition of MLKL significantly attenuates 6K-associated cellular damage and inflammatory release, establishing MLKL-dependent execution as a key modifiable node in viroporin-induced host injury. We further identify HYH09-D4 as a protective small molecule that dampens MLKL activation and limits tissue damage associated with 6K expression. Together, these findings define WEEV 6K as a host-facing viroporin that links ionic dysregulation to MLKL-dependent necroptosis and suggest that targeting MLKL may represent a broadly applicable strategy to mitigate host injury driven by 6K-encoding alphaviruses.

## Introduction

Viral pathogenesis is not determined only by replication burden. Increasing evidence indicates that small viral membrane proteins can disproportionately rewire host physiology by altering membrane ion permeability and electrochemical gradients [1–3]. Virus-encoded ion channels (viroporins) oligomerize to form conductive pores that reshape ion homeostasis, organelle function, and stress-responsive signaling, with consequences that can extend beyond assembly [4–6] and egress to cellular injury and inflammatory escalation [7]. Yet for many zoonotic pathogens, viroporins are still discussed mainly as factors that support the viral life cycle, and their capacity to function as direct drivers of host damage remains incompletely defined. Whether viroporins promote regulated cell death programs and inflammatory escalation during neurotropic infection remains an open question.

Western equine encephalitis virus (WEEV) is a highly neurotropic alphavirus maintained in nature through transmission cycles between vertebrate hosts and specific mosquito vectors [8]. In multiple rodent and primate models, WEEV infection causes pronounced neuronal injury accompanied by glial activation and inflammatory cell infiltration, with both virus-intrinsic cytopathicity and host immune responses contributing to neuropathogenesis [9, 10]. Importantly, in viral injury of the central nervous system, pathological outcomes are often determined not only by viral replication but also by the mode of host cell death [11, 12]. Unlike apoptosis, which is comparatively immunologically silent, lytic forms of regulated necrosis are coupled to membrane rupture and the release of damage-associated signals that can rapidly amplify focal injury into broader neuroinflammation, promoting irreversible tissue damage [13, 14]. Thus, identifying viral determinants that govern death-pathway choice and initiate inflammatory cascades is critical for understanding WEEV neuropathogenesis and for defining potential intervention strategies. However, the viral effector factors that shape cell-death programs and drive inflammatory amplification during WEEV infection remain poorly defined.

Within alphaviruses, the 6K protein is a highly conserved small transmembrane protein classically implicated in late-stage assembly and budding [6]. Prior studies have shown that 6K exhibits viroporin-like activity, altering membrane permeability and modulating the efficiency of infectious particle release [15, 16]. In addition, 6K displays membrane-disruptive properties. For example, the Chikungunya virus (CHIKV) 6K protein has been shown to disrupt intracellular membrane systems [17]. Across multiple alphavirus systems, including SFV, SINV, and GETV, loss or mutation of 6K markedly reduces virion release and replication, with stronger effects in mammalian cells and clear host dependence [18–21], highlighting an evolutionarily conserved function. Despite its established role in the viral life cycle, it remains unresolved whether 6K has additional host-modulatory functions, including effects on regulated cell death pathways and inflammatory injury that may depend on its ion channel activity. Although 6K has been proposed to display channel-associated features, its channel properties have not been systematically defined, and whether 6K can drive specific death programs and contribute functionally to neurovirulence lacks a clear mechanistic chain and reversibility evidence.

In this study, we define WEEV 6K as an ER-enriched viroporin that perturbs intracellular Ca²⁺ store homeostasis and is associated with cellular injury phenotypes linked to MLKL-dependent necroptotic execution. Together, our work expands the conceptual role of alphaviral 6K beyond a late-stage assembly factor to a potential pathogenic effector module and supports the broader premise that virus-encoded ion channels can shape host cell fate and inflammatory amplification in neurotropic infection. These findings provide a mechanistic foundation for targeting host injury pathways in alphavirus-associated neuroinflammation.

## Results

### WEEV 6K forms a cation channel

**Fig. 1:**
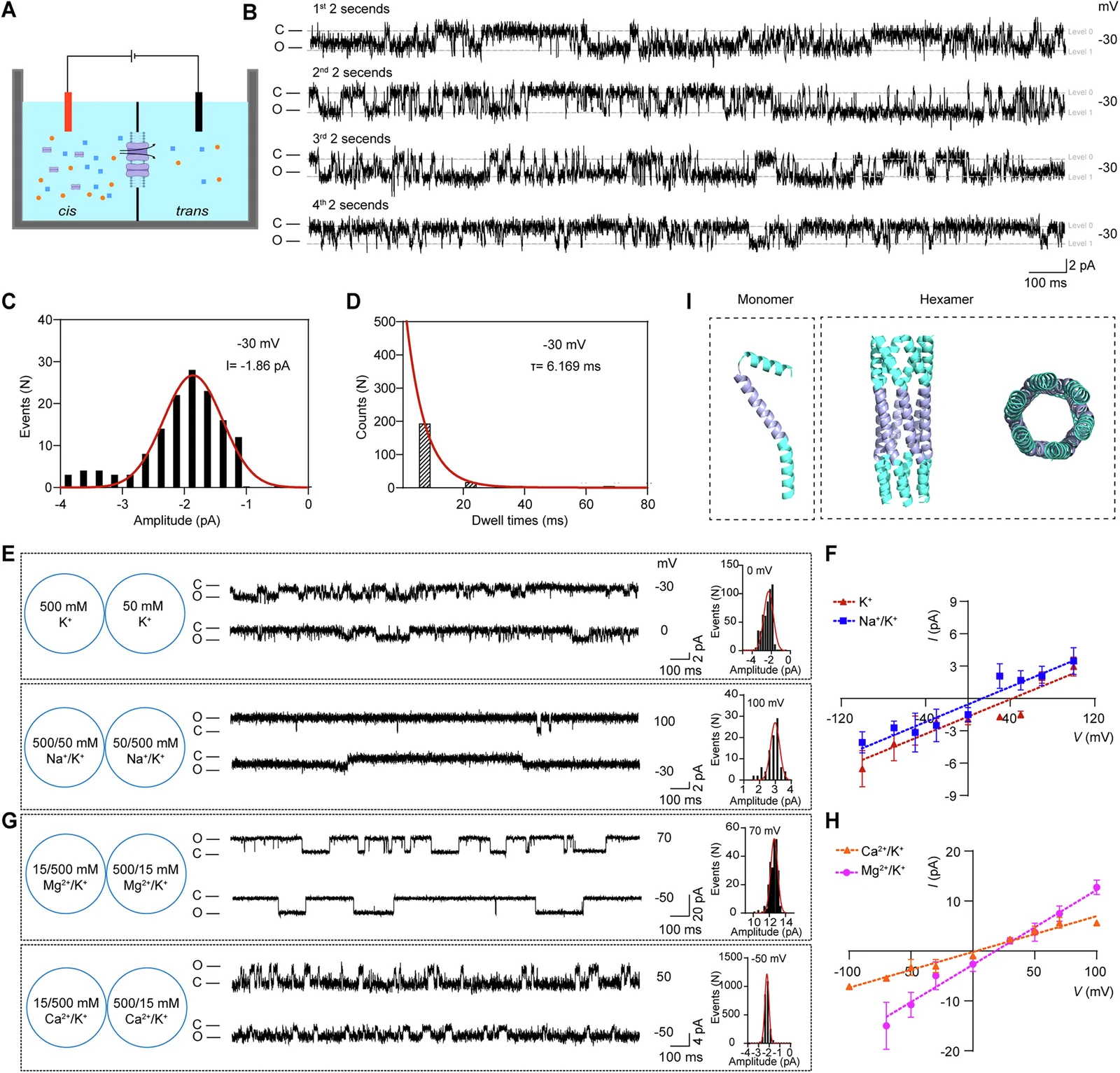
WEEV 6K forms a cation-permeable ion channel. **A** Schematic diagram of the planar lipid bilayer (BLM) recording system. Different colored blocks represent different ionic species. **B** Continuous single-channel current recordings of 6K at −30 mV under a 500:50 mM KCl (cis:trans) gradient with a PC/PS (3:2) lipid composition. “C” means closed; “O” means open. All-point current histograms (**C**) and open-time distribution (**D**) for the trace in **B**. **E** Representative single-channel currents of 6K recorded at 0 mV and −30 mV in K⁺ solution (upper panel), and at 100 mV and −30 mV in mixed K⁺/Na⁺ solution (lower panel). Left schematics illustrate the planar lipid bilayer workstation and the recording solutions on both sides, while the right panels show the corresponding current amplitude histograms. **F** I–V curve of 6K under the solution conditions shown in **E** (*n* = 4 independent experiments). **G** Representative single-channel currents of 6K recorded at 70 mV and −50 mV in mixed K⁺/Mg²⁺ solution (upper panel), and at 50 mV and −50 mV in mixed K⁺/Ca²⁺ solution (lower panel). The left schematic illustrates the planar lipid bilayer workstation and the recording solutions on both sides, while the right panels show the corresponding current amplitude histograms. **H** I–V curve of 6K under the solution conditions shown in **G** (*n* = 4 independent experiments). **I** Hexameric structure of 6K predicted by AlphaFold3 and visualized using PyMOL. Predicted transmembrane regions are highlighted in purple.

To systematically define the channel properties of WEEV 6K, we performed single-channel recordings in planar lipid bilayers (Fig. 1A). Under asymmetric KCl solutions (Cis:Trans = 500:50 mM), addition of purified 6K (Supplementary Fig. S1) to PC/PS (3:2) bilayers produced discrete stepwise current transitions (Fig. 1B), indicating that 6K incorporates into the membrane and forms a conductive pore. Continuous single-channel recording revealed a well-resolved conductance state with consistent current amplitudes at fixed voltages, consistent with a relatively uniform conductive assembly (Fig. 1B). Under a holding potential −30 mV, channel activity remained stable and was dominated by a high open probability with sustained openings (Fig. 1C, D), supporting stable gating behavior in this lipid environment. The single-channel I–V relationship was well described by a linear fit over this voltage range, yielding a slope conductance of 40.12 pS (Fig. 1E). Under the asymmetric KCl solutions, the fitted reversal potential was +41.9 mV (Fig. 1F). This positive reversal potential is close to a cation-selective channel and indicates that the 6K channel conducts predominantly cations under these conditions, with an apparent preference for K⁺ over Cl⁻ (*P*_K⁺_/*P*_Cl_^-^≈8.33). We next examined ion selectivity using asymmetric ion substitutions. In mixed K⁺/Na⁺ solutions, channels remained stable (Fig. 1E) with a reversal potential of +14.3 mV (Fig. 1F), indicating appreciable Na⁺ permeation. In the presence of Ca²⁺ or Mg²⁺, discrete single-channel events remained readily detectable (Fig. 1G, H), and the reversal potential exhibited a negative shift compared with the KCl gradient condition, supporting divalent cation permeation through the 6K channel (*P*_K⁺_/*P*_Ca2+_≈1.18, *P*_K⁺_/*P*_Mg2+_≈0.7). In parallel, AlphaFold3 modeling suggested an oligomeric pore-like assembly of 6K (Fig. 1I), providing structural plausibility for the conductive state observed in bilayers. Together, these data show that WEEV 6K forms a cation channel.

### 6K channel localizes to the ER and reduces Ca^2+^ store release

**Fig. 2:**
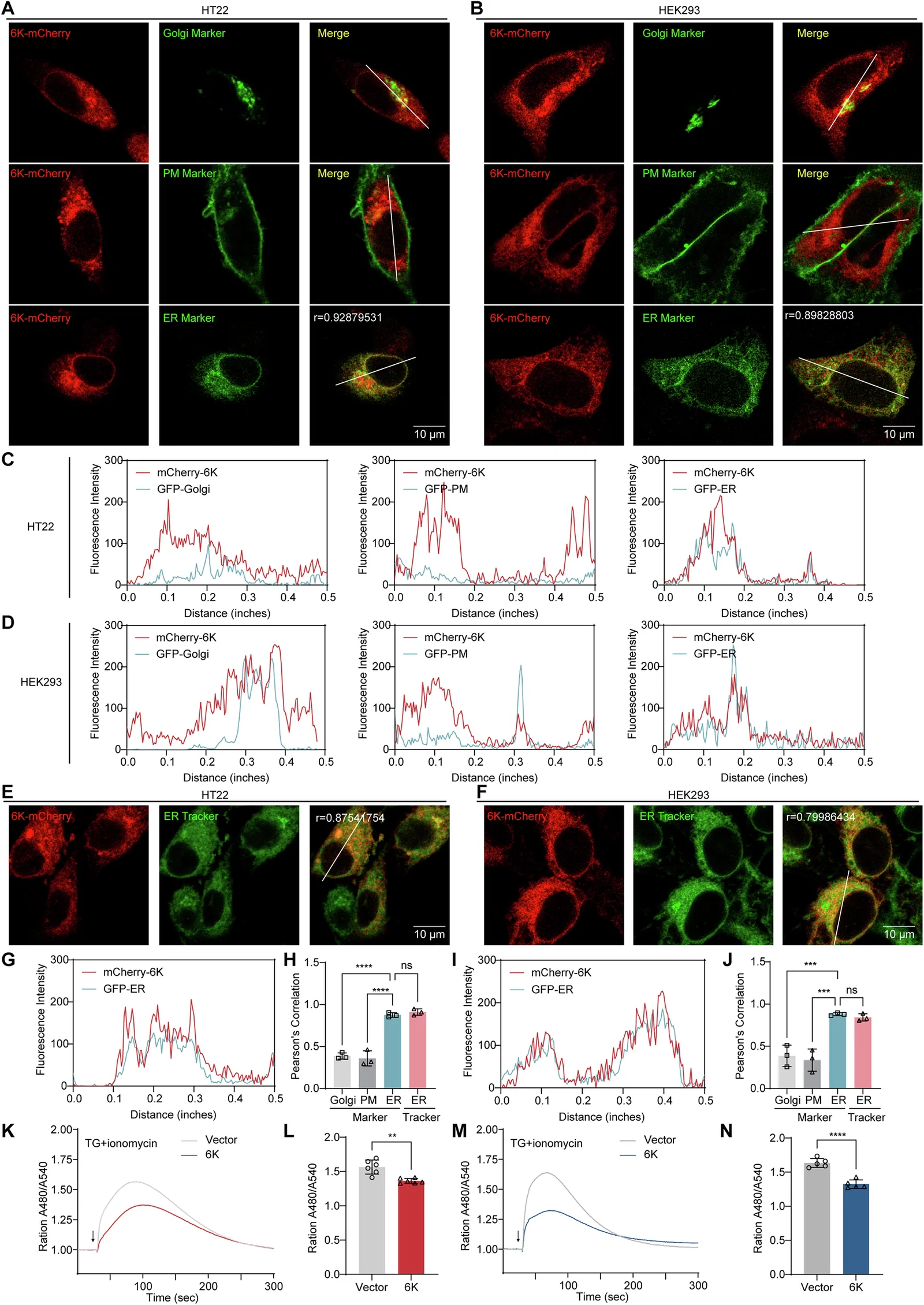
WEEV 6K localizes to the endoplasmic reticulum and disrupts intracellular Ca^2+^ homeostasis. Representative confocal images of HT22 (**A**) and HEK293 (**B**) cells co-transfected with 6K-mCherry (red) and GFP-labeled markers for Golgi, plasma membrane (PM), or endoplasmic reticulum (ER, green) 24 h post-transfection. Colocalization was quantified using Pearson’s correlation coefficient (see **H**–**J**). Scale bars, 10 μm. Representative line-scan profiles showing the fluorescence intensity of 6K-mCherry and GFP-labeled Golgi, PM, or ER markers along the indicated lines in HT22 (**C**) and HEK293 (**D**) cells. Representative images of HT22 (**E**) and HEK293 (**F**) cells 24 h post 6K transfection, stained with ER-Tracker for colocalization analysis. Scale bars, 10 μm. Line-scan profiles showing fluorescence intensity of 6K-mCherry and ER-Tracker along the indicated lines in HT22 (**G**) and HEK293 (**I**) cells. Pearson’s correlation coefficients quantifying colocalization of 6 K with Golgi, PM, ER markers, and ER-Tracker in HT22 (**H**) and HEK293 (**J**) cells, *n* = 3. ^***^p < 0.001, ^****^p < 0.0001, ns not significant, one-way ANOVA. FDSS (fluorometric drug screening system) analysis of evoked intracellular Ca²⁺ release from intracellular stores in HT22 (**K**) and HEK293 (**M**) cells 24 h after 6K transfection. Thapsigargin (TG) and ionomycin (final concentration 10 μM) were added at 30 s (arrows) to induce Ca²⁺ release. **L**, **N** Quantification of Ca²⁺ responses shown in **K** and **M** Data represent mean ± SEM, *n* = 5–6; ^**^p < 0.01, ^****^p < 0.0001, Student’s *t*-test.

To identify the cellular membrane compartment most relevant to 6K channel function, we performed localization analyses in HEK293 cells and HT22 hippocampal neuronal cells. 6K-mCherry was co-transfected with ER-EGFP, Golgi-EGFP, or plasma membrane-EGFP, followed by confocal imaging at 24 h (Fig. 2A, B). In both cell types, 6K displayed a perinuclear, reticular membrane pattern highly consistent with the ER network, whereas overlap with Golgi or plasma membrane markers was weak and largely focal (Fig. 2A, B). Quantitative co-localization confirmed this preference: Pearson’s correlation coefficients were high for ER co-localization (HT22 *r* = 0.93; HEK293 *r* = 0.88) and significantly lower for Golgi or plasma membrane markers. Line-scan profiles further showed near-perfect alignment of fluorescence peaks between 6K and ER markers, with clear offsets relative to Golgi/plasma membrane signals (Fig. 2C, D). ER-Tracker staining provided an independent validation, showing strong spatial overlap between ER-Tracker signals and 6K-mCherry in both backgrounds (Fig. 2E, F, G–J). Given that the ER is the dominant intracellular Ca²⁺ reservoir, we next tested whether 6K alters ER Ca²⁺ store availability using live-cell Ca²⁺ imaging. Upon stimulation with thapsigargin (10 μM) or ionomycin (10 μM), 6K-expressing cells exhibited markedly blunted cytosolic Ca²⁺ transients compared to controls, with reduced peak amplitudes and decreased overall Ca²⁺ release (Fig. 2K–N), reproducible in both HEK293 and HT22 cells. Importantly, this phenotype is most consistent with a reduction in mobilizable ER Ca²⁺ store capacity (i.e., reduced store content and/or impaired loading) rather than nonspecific Ca²⁺ leakage. Collectively, 6K is enriched at the ER and coincides with a reduction in evoked Ca²⁺ release from intracellular stores.

### 6K channel induces necroptosis and promotes cellular injury

**Fig. 3:**
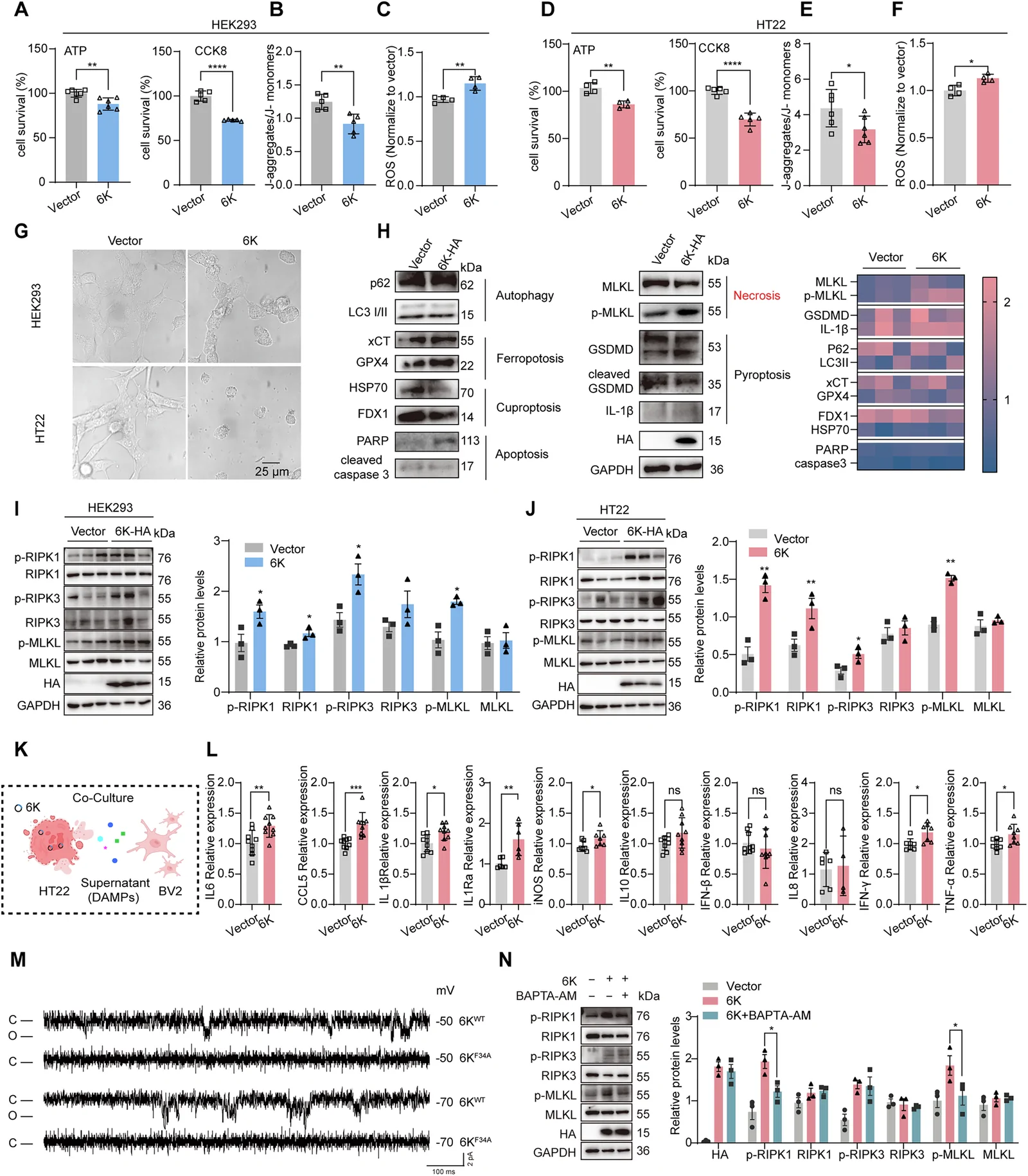
6K induces necroptotic cell death and secondary inflammatory signaling. HEK293 (**A**) and HT22 (**D**) cells were transfected with 6K for 24 h, followed by assessment of cell viability using CCK-8 assay and measurement of intracellular ATP levels. Mitochondrial membrane potential (**B**, **E**) and intracellular reactive oxygen species (ROS) levels (**C**, **F**) were analyzed in HEK293 and HT22 cells 24 h post-6K transfection. Data are presented as mean ± SEM; *n* = 4–6; ^*^p < 0.05, ^**^p < 0.01, Student’s *t*-test. **G** Representative phase-contrast images of HEK293 and HT22 cells 24 h post-transfection with vector or 6K. Scale bar = 25 μm. Immunoblot analysis of cell death pathway markers in HEK293 (**H**) cells transfected with vector or 6K. Markers analyzed include autophagy (p62, LC3 I/II), ferroptosis (xCT, GPX4), cuproptosis(FDX1, HSP70), apoptosis (cleaved caspase 3, PARP), necroptosis (MLKL, p-MLKL), and pyroptosis (GSDMD, cleaved GSDMD, IL-1β). Heatmap represents relative protein expression normalized to GAPDH. Data are presented as mean ± SEM; *n* = 3, Student’s *t*-test. Immunoblot analysis of RIPK1, p-RIPK1, RIPK3, p-RIPK3, MLKL and p-MLKL in HEK293 (**I**) and HT22 (**J**) cells 24 h post-transfected with vector or 6K. GAPDH was used as a loading control. Each lane represents an independent biological replicate derived from separately transfected samples. Densitometric quantification is shown on the right, with grey bars representing the vector control group and blue bars (HEK293) or red bars (HT22) representing the 6K-HA expression group. Data are presented as mean ± SEM; *n* = 3; ^*^p < 0.05, ^**^p < 0.01, Student’s *t*-test. **K** Schematic of the co-culture system. HT22 cells expressing 6K release damage-associated molecular patterns (DAMPs) into the culture supernatant, which was subsequently applied to BV2 microglia. Cytokine expression was assessed after 3 h of incubation. **L** Quantitative PCR analysis of inflammatory cytokine transcripts in BV2 cells following a 3-h exposure to conditioned medium derived from HT22 cells expressing either empty vector or 6K for 24 h. The mRNA levels of IL-6, CCL5, IL-1β, IL-12, iNOS, IL-10, IFN-β, IFN-γ, and TNF-α were measured and normalized to GAPDH. Data are presented as mean ± SEM; *n* = 6; ^*^p < 0.05, ^**^p < 0.01, ^***^p < 0.001, ns not significant, Student’s *t*-test. **M** Representative single-channel currents of 6K^WT^ and 6K^F34A^ recorded at −70 mV and −50 mV under a 500:50 mM KCl (cis:trans) gradient with a PC/PS (3:2) lipid composition. “C” means closed; “O” means open. **N** Immunoblot analysis (left) and densitometric quantification (right) of p-RIPK1, RIPK1, p-RIPK3, RIPK3, MLKL and p-MLKL in HT22 cells transfected with 6K. Cells were treated with BAPTA-AM (1 μM) 6 h post-transfection, and protein levels were analyzed 24 h after transfection. GAPDH was used as a loading control. Data are presented as mean ± SEM; *n* = 3; ^*^p < 0.05, Student’s *t*-test.

Given that the ER localization of 6K and altered Ca²⁺ mobilization, we next asked whether 6K expression is accompanied by broader perturbations of cellular homeostasis (Fig. 3A–F). In both HEK293 and HT22 cells, 6K expression significantly reduced cell viability and decreased intracellular ATP levels (Fig. 3A, D), consistent with reduced energetic capacity. In parallel, mitochondrial membrane potential was markedly reduced (Fig. 3B, E) and ROS levels were increased (Fig. 3C, F), indicating that 6K expression is accompanied by mitochondrial depolarization and accumulation of oxidative stress. This injury phenotype prompted us to define the death program engaged by 6K. In parallel, morphological analysis revealed that 6K expression induced cell rounding and extrusion of cytoplasmic contents (Fig. 3G). We next examined canonical markers across major regulated death pathways to determine the predominant program. Immunoblotting showed that 6K expression significantly increased phosphorylation of MLKL (p-MLKL), the executioner of necroptosis (Fig. 3H). In contrast, canonical markers of apoptosis (PARP, cleaved caspase-3), ferroptosis (GPX4, xCT), pyroptosis (GSDMD, IL-1β), autophagy (p62, LC3-I/II), and cuproptosis-associated factors (HSP70, FDX1) did not exhibit changes that tracked with the injury phenotype (Fig. 3H). We next assessed upstream necroptotic kinase activation and found that 6K increased phosphorylation of RIPK1 and RIPK3 together with concordant elevation of p-MLKL in both HEK293 and HT22 cells (Fig. 3I, J). Together, these results support necroptosis as the major death program engaged by 6K, with activation of RIPK1–RIPK3–MLKL signaling. We next tested whether 6K-driven injury in neuronal-like cells is sufficient to elicit microglial inflammatory activation. Conditioned medium collected from HT22 cells expressing 6K for 24 h was applied to BV2 microglia for 3 h (Fig. 3K). qRT-PCR analyses revealed robust upregulation of pro-inflammatory transcripts, including IL-6, CCL5, IL-1β, and iNOS (Fig. 3L). These data indicate that 6K-associated injury is sufficient to trigger a pro-inflammatory transcriptional program in microglia within a short time window. Together, 6K channels induce a pronounced injury phenotype and engages necroptosis signaling, with conditioned media sufficient to activate microglial pro-inflammatory transcription. Additional mechanistic analyses further support a causal link between 6K channel function, calcium dysregulation, and necroptotic signaling. Structure-guided mutagenesis of the 6K transmembrane domain, including F18A, W20A, C24A, F34A, and R35A, identified F34A as the clearest loss-of-function mutant among the variants tested (Fig. S2A, B). Notably, 6K-F34A lacked detectable channel activity (Fig. 3M) and failed to efficiently induce cell death or phosphorylation of RIPK1, RIPK3, and MLKL (Fig. S2C). In parallel, 6K expression depleted ER calcium stores, whereas intracellular Ca²⁺ buffering with BAPTA-AM attenuated both cytotoxicity (Fig. S2D, E) and activation of the RIPK1-RIPK3-MLKL axis (Fig. 3N). By contrast, inhibition of IP₃R-dependent ER calcium release with 2-APB did not rescue calcium dysregulation, cell death, or necroptotic signaling (Fig. S3). Together, these findings support a model in which 6K perturbs ER calcium homeostasis primarily through its own channel activity and thereby promotes necroptotic pathway activation.

### MLKL depletion suppresses 6K channel driven injury and inflammatory

**Fig. 4:**
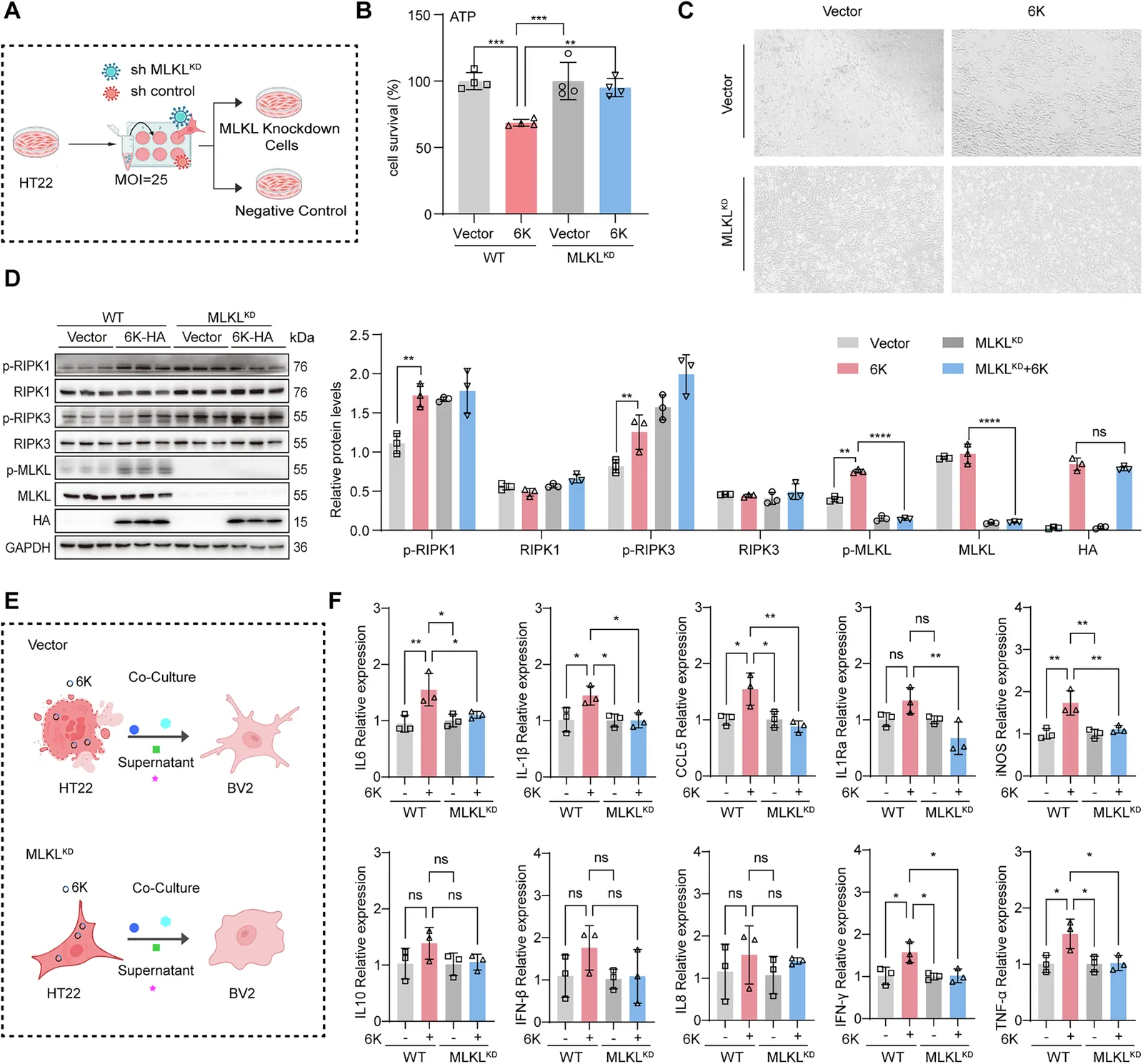
MLKL mediates 6K-induced necroptosis and microglial inflammatory activation. **A** Schematic of MLKL knockdown in HT22 cells using shRNA lentiviral transduction. **B**, **C** Cell viability of HT22 cells expressing 6K for 24 h in the presence or absence of MLKL knockdown. Viability was assessed by intracellular ATP measurement (**B**) and representative cell morphology imaging (**C**). Data are presented as mean ± SEM; *n* = 4; ^**^p < 0.01, ^***^p < 0.001, one-way ANOVA. **D** Immunoblot analysis of RIPK1, p-RIPK1, RIPK3, p-RIPK3, MLKL, p-MLKL, HA and GAPDH in wild-type (WT) and MLKL knockdown (MLKL^KD^) HT22 cells transfected with vector or 6K-HA. GAPDH was used as a loading control. Each lane represents an independent biological replicate derived from separately transfected samples. The experimental groups are defined as follows: WT cells with Vector (light grey), WT cells with 6K (red), MLKL^KD^ cells with Vector (dark grey), and MLKL^KD^ cells with 6K (blue). Statistical analysis is shown on the Right. Data are presented as mean ± SEM; *n* = 3; ^**^p < 0.01, ^****^p < 0.0001, ns not significant, one-way ANOVA. **E** Schematic of the conditioned medium transfer assay. Supernatants from 6K-expressing HT22 cells (WT or MLKL^KD^) were collected and applied to BV2 microglia to assess inflammatory responses. **F** Quantitative PCR analysis of pro-inflammatory cytokine transcripts in BV2 cells exposed to conditioned medium. Relative expression was normalized to GAPDH. Data are presented as mean ± SEM; *n* = 6; ^*^p < 0.05, ^**^p < 0.01, ns not significant, one-way ANOVA.

To test whether MLKL contributes to 6K-driven necroptosis and cellular injury, we depleted MLKL in HT22 cells using shRNA lentiviral transduction (Fig. 4A), with efficient delivery confirmed by GFP fluorescence (Fig. S4). We then expressed 6K in the MLKL knockdown background and quantified cell viability. Compared to control cells, MLKL depletion significantly attenuated 6K-associated cell death and partially restored viability (Fig. 4B, C), indicating that the cytotoxic phenotype elicited by 6K depends on MLKL. We next examined how MLKL depletion reshapes the necroptotic signaling cascade. In control cells, 6K expression was accompanied by increased phosphorylation of RIPK1 and RIPK3 together with elevated p-MLKL (Fig. 4D), consistent with activation of the canonical RIPK1–RIPK3–MLKL pathway. In contrast, under MLKL knockdown conditions, 6K failed to induce significant changes in RIPK1, RIPK3, or their phosphorylation states (Fig. 4D), consistent with impaired transmission of necroptotic signaling downstream of 6K. Notably, empty-vector transfection alone caused a modest increase in basal p-RIPK1 and p-RIPK3 relative to untreated cells, suggesting that the elevated basal phosphorylation observed under MLKL knockdown likely reflects, at least in part, transfection-associated stress rather than a specific consequence of MLKL depletion (Fig. S5). Subtle feedback or compensatory effects of MLKL loss, however, cannot be fully excluded. Given that necroptotic execution can promote release of inflammatory cues, we next assessed microglial inflammatory responses using an HT22–BV2 conditioned-medium assay (Fig. 4E). Supernatants from 6K-expressing control HT22 cells induced pro-inflammatory transcripts in BV2 microglia, whereas supernatants derived from MLKL-depleted HT22 cells significantly reduced induction of IL-6, IL-1β, CCL5, iNOS, IFN-γ, and TNF-α (Fig. 4F). These results are consistent with MLKL-dependent necroptotic execution contributing to microglial inflammatory activation downstream of 6K-induced injury.

### Targeting MLKL identifies HYH09-D4 as a protective modulator of 6K-mediated injury

**Fig. 5:**
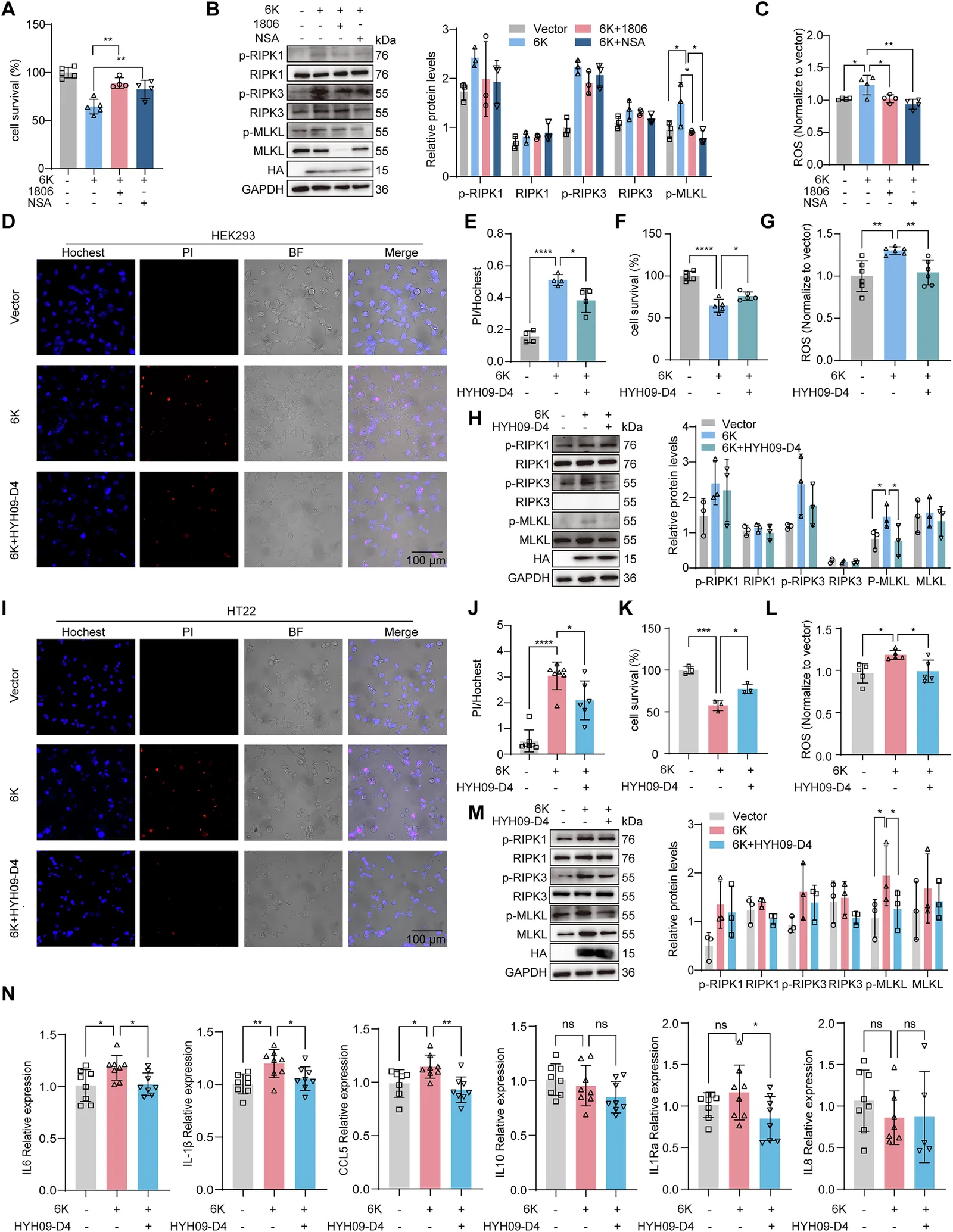
Pharmacological inhibition of MLKL attenuates 6K-induced necroptosis and inflammatory signaling. Intracellular ATP levels (**A**) and reactive oxygen species (ROS) levels (**C**) in HEK293 cells were measured 24 h post-transfection. Cells were transfected with 6K and treated with NSA (10 μM) or 1806 (10 μM) 6 h post-transfection. Data are presented as mean ± SEM; *n* = 4–5; ^*^p < 0.05, ^**^p < 0.01, one-way ANOVA. **B** Immunoblot analysis (left) and densitometric quantification (right) of p-RIPK1, RIPK1, p-RIPK3, RIPK3, MLKL and p-MLKL in HEK293 cells transfected with 6K. Cells were treated with NSA (10 μM) or 1806 (10 μM) 6 h post-transfection, and protein levels were analyzed 24 h after transfection. GAPDH was used as a loading control. Data are presented as mean ± SEM; *n* = 3; ^*^p < 0.05, Student’s *t*-test. **D**, **E**, **I**, **J** Cell death in HEK293 (**D**) and HT22 (**I**) cells was assessed 24 h post-transfection by PI/Hoechst double staining. Scale bar = 100 μm. Quantification is shown on the right (**E**, **J**). Data are presented as mean ± SEM; *n* = 4; ^*^p < 0.05, ^****^p < 0.0001, one-way ANOVA. Intracellular ATP levels and ROS levels in HEK293 (**F**, **G**) and HT22 (**K**, **L**) cells were measured 24 h post-transfection. Cells were transfected with 6K and treated with HYH09-D4 (10 μM) 6 h post-transfection. Data are presented as mean ± SEM; n = 3-6; ^*^p < 0.05, ^**^p < 0.01, ^***^p < 0.001, one-way ANOVA. Immunoblot analysis (left) and densitometric quantification (right) of p-RIPK1, RIPK1, p-RIPK3, RIPK3, MLKL and p-MLKL in HEK293 (**H**) and HT22 (**M**) cells transfected with 6K. Cells were treated with HYH09-D4 (10 μM) 6 h post-transfection, and protein levels were analyzed 24 h after transfection. GAPDH was used as a loading control. Data are presented as mean ± SEM; *n* = 3; ^*^p < 0.05, Student’s *t*-test. **N** Quantitative PCR analysis of pro-inflammatory cytokine transcripts in BV2 cells. Relative expression was normalized to GAPDH. Data are presented as mean ± SEM; *n* = 8; ^*^p < 0.05, ^**^p < 0.01, ns not significant, one-way ANOVA.

Given the genetic evidence implicating MLKL in 6K-associated necroptotic injury, we next asked whether pharmacological targeting of MLKL can mitigate 6K-driven cytotoxicity and enable identification of protective small molecules. In HEK293 cells overexpressing 6K, treatment with the MLKL inhibitor necrosulfonamide (NSA, 10 μM) or the MLKL-targeting PROTAC degrader 1806 (10 μM) significantly improved cell viability and reduced ROS accumulation, indicating that pharmacological inhibition of MLKL can partially attenuate 6K-associated injury. Immunoblotting further showed that both NSA and 1806 reduced p-MLKL levels without appreciably altering RIPK1 or RIPK3 abundance or phosphorylation of RIPK1 and RIPK3, consistent with preferential modulation of the MLKL execution step rather than upstream kinase expression and activation (Fig. 5A–C). We then performed a pilot screen of 47 in-house compounds using intracellular ATP as a viability readout and identified HYH09-D4 as a reproducible protective hit in both HEK293 and HT22 cells. In validation assays, HYH09-D4 (10 μM) significantly reduced 6K-associated cell death and restored ATP levels, accompanied by a decrease in PI-positive cells in PI/Hoechst staining and partial normalization of nuclear morphology (Fig. 5D–H, I–L). HYH09-D4 also partially reduced 6K-associated ROS elevation (Fig. 5G, L). Mechanistically, HYH09-D4 decreased p-MLKL levels while leaving the total abundance and phosphorylation of RIPK1 and RIPK3 largely unchanged (Fig. 5H, M), suggesting that its protective activity is linked to suppression of MLKL activation and/or downstream execution events.

Because 6K-associated necroptotic injury drives inflammatory spillover, we further tested whether HYH09-D4 modulates microglial inflammatory responses in an HT22–BV2 conditioned-medium paradigm. Supernatants from HYH09-D4-treated, 6K-expressing HT22 cells significantly reduced the induction of pro-inflammatory transcripts in BV2 microglia (Fig. 5N), indicating that pharmacological attenuation of the MLKL execution step can dampen both cell-autonomous injury and secondary inflammatory output. Together, targeting of MLKL reduces p-MLKL and partially mitigates 6K-associated injury and inflammatory signaling. HYH09-D4 provides a useful chemical probe to interrogate MLKL-dependent injury pathways downstream of 6K.

## Discussion

Our study defines the ion channel properties of the WEEV 6K protein and links its host-facing activity to cellular injury and inflammatory outcomes. We show that 6K forms a functional ion channel with broad cation permeability in a defined membrane system and that its expression in cells is associated with injury phenotypes accompanied by activation of MLKL-dependent necroptotic signaling. Importantly, 6K-driven cellular damage can be effectively attenuated through genetic deletion or pharmacological inhibition of MLKL, identifying necroptotic execution as a modifiable step in 6K-associated injury. Beyond cell-intrinsic effects, conditioned signals from 6K-expressing neuronal-like cells elicit a pro-inflammatory transcriptional response in microglia-like cells, indicating that 6K activity can influence local inflammatory responses. Together, these findings suggest that a virus-encoded ion channel can contribute directly to host injury and inflammation and highlight MLKL as a potential intervention point in infections involving 6K-encoding alphaviruses.

Viroporins are widely recognized to perturb host electrochemical gradients and promote membrane remodeling, assembly, and egress [4, 5, 22], and deletion of channel-encoding genes can markedly reduce progeny production [6]. As a result, these proteins have largely been viewed through the lens of viral replication, whereas their potential to directly contribute to host injury has received less attention. Our findings indicate that the pathogenic consequences of viroporin activity depend not only on the presence of channel function but also on the ionic species conducted and the intracellular membranes at which channel activity is deployed. In this regard, viroporins that conduct Ca²⁺ are of particular interest. Although many viral ion channels preferentially conduct protons or monovalent cations [23, 24], several well-characterized viroporins, including picornavirus 2B and rotavirus NSP4, conduct Ca²⁺, disrupt Ca²⁺-dependent signaling, and promote regulated cell death and inflammasome activation [25–27]. Ca²⁺ is a central second messenger for stress adaptation, regulated death, and inflammatory programs, and viroporin-driven Ca²⁺ dysregulation is widely viewed as an early trigger for multiple death modalities [28]. The ER is the major intracellular Ca²⁺ reservoir and a key determinant of stress thresholds [29]; accordingly, ER-localized viroporins may alter Ca²⁺ reserves and bias downstream signaling [30, 31]. Consistent with this view, WEEV 6K displays divalent cation permeability, localizes preferentially to ER membranes, and its expression is associated with impaired mobilization of intracellular Ca²⁺ stores. Importantly, buffering intracellular calcium with BAPTA-AM nearly abolished 6K-induced necroptotic signaling and cell death, establishing calcium flux as an indispensable upstream trigger. Furthermore, the failure of IP₃R inhibition to mitigate this injury confirms that 6K operates as an autonomous pore rather than merely hyperactivating host ER calcium channels. Together, these observations support a model in which 6K directly disrupts ER Ca²⁺ homeostasis to promote cellular stress states that favor entry into MLKL-dependent necroptotic signaling. Unlike apoptosis, necroptosis culminates in membrane permeabilization and release of damage-associated cues that can promote immune activation [14]. This feature is especially relevant in the CNS, where microglia rapidly respond to injury signals and can amplify inflammation [32]. In an HT22–BV2 conditioned-medium setting, supernatants from 6K-expressing neuronal-like cells induce a pro-inflammatory transcriptional response in microglia-like cells, and this response is substantially reduced when MLKL is depleted, consistent with necroptotic execution shaping inflammatory cue strength.

Our findings indicate that MLKL-dependent necroptotic execution contributes substantially to cellular injury and inflammatory responses associated with 6K expression. Given that tissue damage and inflammation during neurotropic viral infection can arise from host death pathway activation in addition to viral replication, modulation of terminal necroptotic signaling may represent a viable host-directed approach to limit injury. MLKL functions as the terminal effector of necroptosis, directly regulating membrane permeabilization and downstream damage signaling through alterations in membrane ion flux [33, 34]. Notably, although WEEV 6K and MLKL share the capacity for membrane interaction and oligomerization, our sequence and structural analyses do not support direct homology between them. Rather, they likely represent distinct membrane-active proteins that converge functionally on membrane permeabilization (Fig. S6).

Consistent with the importance of this pathway, multiple viruses have evolved mechanisms to interfere with necroptosis, including the expression of MLKL-like proteins that antagonize RIPK3 signaling [35]. Moreover, necroptosis has been implicated in viral neuroinflammation in vivo, including models of herpes simplex encephalitis [36, 37] and Japanese encephalitis virus infection [38]. In the context of 6K overexpression, genetic loss of MLKL reduces cell death and attenuates the pro-inflammatory responses elicited in microglia-like cells by conditioned media from 6K-expressing cultures. In parallel, pharmacological inhibition or targeted degradation of MLKL lowers phosphorylated MLKL levels and partially rescues cell viability and oxidative stress phenotypes associated with 6K expression. These observations indicate that MLKL-dependent execution plays a central role in determining the extent of both cell-intrinsic injury and secondary inflammatory signaling downstream of 6K. Consistent with this conclusion, pharmacological suppression of MLKL activity dampens injury phenotypes across multiple cellular contexts. NSA and the MLKL-directed degrader 1806 reduce MLKL phosphorylation and mitigate 6K-associated damage, while the protective compound HYH09-D4 similarly limits injury and inflammatory responses while decreasing p-MLKL levels. Although the precise mechanism by which HYH09-D4 modulates MLKL activity remains to be defined, its effects support the existence of pharmacologically accessible steps within the MLKL-dependent pathway that can be leveraged to constrain 6K-driven host injury.

Several limitations remain. The contribution and sufficiency of 6K should be established in authentic infection and in vivo, and the structural and lipid-dependent determinants of 6K channel assembly and ion flux directionality warrant further definition. In addition, identifying the direct target and mechanism of HYH09-D4 will be important for clarifying its utility and translational potential. Despite these limitations, our findings support a broader principle: virus-encoded ion channels can extend beyond lifecycle facilitation to act as host-facing effectors that shape regulated death and inflammatory amplification, and they can expose tractable intervention points at the level of necroptotic execution.

## Materials and methods

### Plasmids and compounds

The wild-type WEEV 6K coding sequence was synthesized by Tsingke Biotechnology Co., Ltd (China). For recombinant protein expression and purification, the 6K sequence was cloned into the pGEX-6P1 vector with a C-terminal His-tag (pGEX-6P1-GST-PPase-6K-8his). For cell-based assays, pcDNA3.1 vectors were used for cell viability analysis and fluorescence imaging experiments. All compounds used for screening were obtained from the Chinese National Compound Library (CNCL). HYH09-D4 refers to a compound from the HYH series, plate 09, well D4.

### Protein purification

The pGEX-6P1-GST-PPase-6K-8his plasmid was transformed into E. coli BL21 (DE3) pLysS competent cells (TransGen Biotech, China, Cat# CD701-02) and cultured in 50 mL LB medium supplemented with 50 μg/mL ampicillin (Yeasen, China, Cat# 60203ES60). The culture was then diluted 1:100 into 1 L LB medium and grown at 37 °C with shaking at 220 rpm until the optical density at 600 nm (OD_600_) reached approximately 0.6–0.8. Protein expression was induced by adding 0.5 mM IPTG (Yeasen, China, Cat# 10902ES60), and the culture was incubated at 18 °C with shaking at 180 rpm for 16–20 h. After induction, cells were harvested by centrifugation and resuspended in lysis buffer containing 150 mM NaCl, 20 mM Tris-HCl (pH 8.0), 1 mM phenylmethylsulfonyl fluoride (PMSF) and 1× EDTA-free protease inhibitor cocktail (Yeasen, China, Cat# 20123ES50). Cells were lysed using a high-pressure homogenizer at 700 bar for 6 min at 4 °C. The lysate was centrifuged at 12,000 × *g* for 30 min at 4 °C to remove insoluble debris. The clarified supernatant was filtered through a 0.22 μm membrane and subsequently incubated with 1% (w/v) n-dodecyl-β-D-maltoside (DDM; Sinopharm, China, Cat# XW016922793603) for 1 h at 4 °C with gentle rotation to solubilize membrane-associated proteins. Following detergent solubilization, the supernatant was loaded onto Ni-NTA resin (Yeasen, China, Cat# 20502ES60) pre-equilibrated with TBS buffer (150 mM NaCl, 20 mM Tris-HCl, pH 8.0) and incubated for 1 h at 4 °C with gentle rotation for affinity binding. After binding, the resin was sequentially washed with TBS buffer containing 20, 30, and 50 mM imidazole (10 column volumes each; all buffers were supplemented with 0.03% DDM), and the protein was eluted with TBS buffer containing 300 mM imidazole (Yeasen, China, Cat# 20501ES76) and 0.03% DDM. Eluted proteins were concentrated using an ultrafiltration device with a 10 kDa molecular weight cutoff and stored at −80 °C for subsequent electrophysiological recordings.

### Planar lipid bilayer (BLM) electrophysiology

Electrophysiological recordings were performed using a Planar Lipid Bilayer Workstation (BLM Workstation). The system separated the solution into two compartments via a 200-μm aperture, with the Cup defined as the cis side and the Chamber as the trans side. To determine the ionic selectivity of the channel, various salt gradients were established as follows: (1) cis: 500 mM KCl, 5 mM HEPES, pH 6.5; trans: 50 mM KCl, 5 mM HEPES, pH 6.5; (2) cis: 500 mM KCl, 50 mM NaCl, 5 mM HEPES, pH 6.5; trans: 50 mM KCl, 500 mM NaCl, 5 mM HEPES, pH 6.5; (3) cis: 500 mM KCl, 15 mM CaCl₂, 5 mM HEPES, pH 6.5; trans: 50 mM KCl, 150 mM CaCl₂, 5 mM HEPES, pH 6.5; (4) cis: 500 mM KCl, 15 mM MgCl₂, 5 mM HEPES, pH 6.5; trans: 50 mM KCl, 150 mM MgCl₂, 5 mM HEPES, pH 6.5.

Phospholipids (phosphatidylcholine:phosphatidylserine = 3:2) dissolved in decane were carefully painted over the aperture in the Cup to form a stable lipid bilayer. All lipids were purchased from Avanti Polar Lipids (USA). Purified protein was added to the cis compartment to allow incorporation into the bilayer. Single-channel currents were recorded under voltage-clamp mode using a Warner BC-535 bilayer clamp amplifier (Warner Instruments, USA), filtered at 1–2 kHz, and sampled at 10 kHz. Analog signals were digitized with Digidata 1440A (Molecular Devices, USA) and analyzed using pClamp 10.4 software. Events with durations shorter than 1.5 ms were excluded from analysis. Single-channel conductance was determined by fitting the amplitude distribution to a Gaussian function. Equilibrium potentials were calculated by linear fitting of the current–voltage (I–V) relationships. Ion permeability ratios were estimated using the Goldman–Hodgkin–Katz (GHK) flux equation.

$${\rm{For}}\,{\rm{monovalent}}\,{\rm{ion}}:Erev=\frac{RT}{F}\,{\mathrm{ln}}\frac{P{X}^{+}[{X}^{+}]out+P{Y}^{+}[{Y}^{+}]out}{P{X}^{+}[{X}^{+}]in+P{Y}^{+}[{Y}^{+}]in}$$

$${\rm{For}}\,{\rm{divalent}}\,{\rm{ion}}:Erev=\frac{RT}{F}\,\mathrm{ln}\frac{P{X}^{+}[{X}^{+}]out(1+{e}^{\frac{F\ast Erev}{RT}})+4P{Y}^{2+}[{Y}^{2+}]out}{P{X}^{+}[{X}^{+}]in(1+{e}^{\frac{F\ast Erev}{RT}})+4P{Y}^{2+}[{Y}^{2+}]in{e}^{\frac{F\ast Erev}{RT}}}$$

### Cell culture and transfection

Mouse microglia cells (BV2), Human embryonic kidney cells (HEK293) and Hippocampal neuronal cell line (HT22) were purchased from National Collection of Authenticated Cell Cultures. Both cell lines were maintained in DMEM (Gibco, USA, Cat# 11054001) supplemented with 10% fetal bovine serum (FBS, Gibco, USA, Cat# 10100) at 37 °C in a humidified incubator with 5% CO_2_. Cells were seeded and transfected the following day at approximately 40–60% confluency. Plasmid transfections were performed using Lipofectamine 3000 (Invitrogen, USA, Cat# L3000015) according to the manufacturer’s instructions.

### Confocal laser scanning microscopy

Confocal imaging was performed using a Zeiss LSM 900 inverted fluorescence microscope (Zeiss, Germany). Regions of interest were initially identified with a low-magnification objective, and high-resolution colocalization images were subsequently acquired using a 63× oil-immersion objective. mCherry-tagged proteins were excited with the 552 nm laser line, while EGFP-tagged proteins were excited with the 488 nm laser line.

### FDSS Ca ion fluorescence release assay

Cells were seeded into black, glass-bottom 96-well plates and transfected the following day. Intracellular Ca²⁺ levels were measured 24 h after transfection using the fluorescent calcium indicator Fluo-4 (Invitrogen, USA, Cat# F14217). Prior to measurement, the culture medium was removed, and cells were gently washed once with calcium-free extracellular solution (140 mM NaCl, 5 mM KCl, 1 mM MgCl_2_, 2 mM Probenecid, 10 mM Glucose, 10 mM HEPES, adjusted to pH 7.4 with NaOH). Fluo-4 was diluted 1:1000 in calcium-free solution to a final concentration of 2 μM and incubated with cells at 37 °C in the dark for 1 h. After incubation, cells were washed twice to remove excess dye, and 80 μL of calcium-free extracellular solution was added to each well. Calcium release was stimulated by the addition of thapsigargin (TG,10 μM, CST, USA, Cat# 12758S) and ionomycin (10 μM, MCE, USA, Cat# HY-13434), and intracellular Ca²⁺ fluorescence was recorded in real time in kinetic mode using the FDSS/μCell system (Hamamatsu, Japan).

### Mitochondrial membrane potential assay (JC-1)

HEK293 cells and HT22 cells were seeded into 96-well plates at 8000 cells per well, and transfected with pcDNA3.1-6K or empty vector. Twenty-four hours after transfection, mitochondrial membrane potential was assessed using the JC-1 assay kit (Beyotime, China, Cat# C2006). Carbonyl cyanide 3-chlorophenylhydrazone (CCCP, 10 μM) was used as a positive control. JC-1 working solution was prepared according to the manufacturer’s instructions and added to the cells, followed by incubation at 37 °C in the dark for 30 min. After incubation, cells were washed three times with the JC-1 dilution buffer provided in the kit. Mitochondrial membrane potential changes were quantified by measuring green fluorescence (488/525 nm) and red fluorescence (540/590 nm) using a microplate reader (BioTek Synergy H1, USA) and calculating the red/green fluorescence intensity ratio.

### Intracellular ROS measurement

HEK293 and HT22 cells were seeded into 96-well plates at a density of 8000 cells per well, transfected with pcDNA3.1-6K or empty vector, and analyzed 24 h post-transfection. Intracellular reactive oxygen species (ROS) levels were assessed using a ROS assay kit (Beyotime, China, Cat# S0033S). Rosup (50 mg/mL,1:1000 dilution) was used as a positive control and incubated with cells at 37 °C in the dark for 30 min. For ROS detection, the DCFH-DA probe was diluted in PBS to a final concentration of 10 μM and loaded into cells, followed by incubation at 37 °C in the dark for 30 min. Cells were then washed three times with PBS to remove excess probe. Fluorescence intensity was measured using a fluorescence microplate reader (BioTek Synergy H1, USA) with excitation at 488 nm and emission at 525 nm.

### Cell viability and cytotoxicity assay

Cell viability was assessed using a CCK-8 assay kit (Yeasen, China, Cat# 40203ES60), and absorbance at 450 nm was measured with a microplate reader from Thermo Fisher Scientific (Waltham, MA, USA). Cellular ATP levels were determined using a Cell-ATP Viability Detection kit(MCE, USA, Cat# HY-K0302), and chemiluminescence signals were recorded with a microplate reader (BioTek Synergy H1, USA).

### PI and Hoechst double-staining analysis

After treatment, cells were gently washed once with PBS and stained with Hoechst 33342 (Invitrogen, USA, Cat# R37165) at a final concentration of 1 μM, followed by incubation at 37 °C in the dark for 10–15 min to label nuclei. Propidium iodide (PI) was subsequently added at a final concentration of 1 μM, and cells were incubated at room temperature in the dark for 5–10 min. After staining, cells were gently washed with PBS to remove excess dye and immediately observed and imaged under a fluorescence microscope (Leica Thunder DMi8, Germany). Hoechst 33342 emitted blue fluorescence to label all nuclei, while PI emitted red fluorescence and selectively stained cells with compromised membrane integrity, allowing differentiation between live and dead cells.

### MLKL lentiviral infection

HT22 cells were seeded in 6-well plates and cultured to 20–30% confluency prior to infection. Cells were infected with MLKL-targeting lentivirus (Genechem, China, Cat# 134FE68) at a multiplicity of infection (MOI) of 25 using the infection enhancer provided by the manufacturer. After 16 h of incubation at 37 °C, the viral supernatant was replaced with fresh DMEM supplemented with 10% fetal bovine serum (FBS), and cells were further cultured for 48–72 h to allow effective knockdown of MLKL. Knockdown efficiency was validated by Western blotting. Only cells with confirmed knockdown were used for subsequent experiments. The shRNA sequence targeting mouse MLKL was GGAGATTCCAAAGGAACATCT.

### Western blot assay and antibodies

Cells were collected 24 h after transfection and lysed on ice for 15 min using RIPA lysis buffer supplemented with a protease inhibitor cocktail (Yeasen, China, Cat# 20123ES50). Lysates were centrifuged at 12,000 × *g* for 15 min at 4 °C, and the supernatants were collected. Protein concentrations were determined using the BCA assay (Thermofisher, USA, Cat# A55864). Samples were mixed with 5× SDS loading buffer and denatured at 95 °C for 10 min. Proteins were separated by 10% SDS-PAGE and transferred onto PVDF membranes. Membranes were blocked with 5% skim milk and incubated with primary antibodies overnight at 4 °C. The secondary antibodies used were peroxidase-conjugated Goat Anti-Rabbit IgG (H+L) (Yeasen, China, Cat# 33101ES60) and peroxidase-conjugated Goat Anti-Mouse IgG (H+L) (Yeasen, China, Cat# 33201ES60).

For HEK293 cells, antibodies against GAPDH (Yeasen, China, Cat# 30201ES20), HA-Tag (CST, USA, Cat# 3724S), p62 (CST, USA, Cat# 5114S), LC3 I/II (CST, USA, Cat# 12741T), xCT (ABclonal, China, Cat# A2413), GPX4 (CST, USA, Cat# 52455T), HSP70 (CST, USA, Cat# 4876T), FDX1 (ABclonal, China, Cat# A20895), PARP (CST, USA, Cat# 5625T), Cleaved caspase-3 (CST, USA, Cat# 9664), GSDMD (CST, USA, Cat# 39754S), IL-1β (ABclonal, China, Cat# A16288), MLKL (Abcam, UK, Cat# ab184718), phosphorylated MLKL (p-MLKL; Abcam, UK, Cat# ab187091), RIPK1 (CST, USA, Cat# 3493S), phosphorylated RIPK1 (p-RIPK1; CST, USA, Cat# 65746 T), RIPK3 (CST, USA, Cat# 10188T) and phosphorylated RIPK3 (p-RIPK3; CST, USA, Cat# 93654T) were used. For HT22 cells, antibodies against GAPDH (Yeasen, China, Cat# 30201ES20), HA-Tag (CST, USA, Cat# 3724S), MLKL (CST, USA, Cat# 26539S), p-MLKL (CST, USA, Cat# 37333S), RIPK1 (CST, USA, Cat# 3493S), p-RIPK1 (CST, USA, Cat# 38662S), RIPK3 (CST, USA, Cat# 95702S) and p-RIPK3 (Abclonal, China, Cat# AP1185) were used.

### Quantitative real-time PCR(qRT-PCR) analysis

Cells were collected, and 1 mL of TRIzol (Invitrogen, USA, Cat# 15596026CN) reagent was added to each tube. Cells were thoroughly lysed by pipetting and incubated at room temperature for 3 min. Subsequently, 200 μL of chloroform was added, and the mixture was vigorously shaken for 30 s, followed by centrifugation at 12,000 × *g* for 15 min at 4 °C. The upper aqueous phase (~400 μL) was carefully transferred to a new tube and mixed with an equal volume of isopropanol by inverting the tube 15 times. The mixture was incubated at room temperature for 10 min and then centrifuged at 12,000 × *g* for 15 min at 4 °C. The resulting RNA pellet was washed with 70% ethanol (prepared with DEPC-treated water) and centrifuged at 12,000 × *g* for 10 min at 4 °C. After discarding the supernatant, the pellet was air-dried and dissolved in RNase-free water. RNA concentration was determined using a Nanodrop spectrophotometer. cDNA was synthesized from extracted RNA using the ABScript III RT Master Mix (Abclonal, China, Cat# RK20429) according to the manufacturer’s instructions. Quantitative PCR was performed using SYBR Green Master Mix (Yeasen, China, Cat# 11184ES03) on a CFX384 real-time PCR system (Bio-Rad). GAPDH was used as an internal control. Relative expression levels were calculated using the 2^−ΔΔCt^ method. The primer sequences used for quantitative analysis are listed in the table below. All primer sequences listed in Table 1 were designed specifically for mouse genes.

**Table 1:** Primer sequences used for qRT-PCR analysis in mouse.

Primer name	FWD sequence	REV sequence
IL-6	AGTTGCCTTCTTGGGACTGA	TCCACGATTTCCCAGAGAAC
IL-1β	GAAGTTGACGGACCCCAAAA	CCACAGCCACAATGAGTGATAC
CCL5	CCCTCACCATCATCCTCACT	CCTTCGAGTGACAAACACGA
IL1-Ra	CCAGCTCATTGCTGGGTACT	TTCTCAGAGCGGATGAAGGT
iNOS	GTTCTCAGGCCAACAATACAAGA	GTGGACGGGTCGATGTCAC
IL-10	ATAACTGCACCCACTTCCCA	GGGCATCACTTCTACCAGGT
IFN-β	ACCTACAGGGCGGACTTCAA	GTCTCATTCCACCCAGTGCT
IL-8	CGTCCCTGTGACACTCAAGA	TAATTGGGCCAACAGTAGCC
IFN-γ	TCCTGCACCAACATTTCTGA	TACGAGGACGGAGAGCTGTT
TNF-α	TCGTAGCAAACCACCAAGTG	GGAGTAGACAAGGTACAACCCA
GAPDH	TGGCCTTCCGTGTTCCTAC	GAGTTGCTGTTGAAGTCGCA

### Statistical analysis

All statistical analyses were performed using GraphPad Prism 8 software. Semi-quantitative analysis of Western blot bands was performed using ImageJ software. Statistical differences between two groups were analyzed using a two-sided Student’s *t*-test, whereas one-way ANOVA was utilized for comparisons among multiple groups. All the experiments were performed independently at least three times. Data are presented as mean ± SEM. Statistical significance was defined as ^*^p < 0.05, ^**^p < 0.01, and ^***^p < 0.001.

## Supplementary information

Supplementary Information

Supplementary Information- Figure S1

Supplementary Information- Figure S2

Supplementary Information- Figure S3

Supplementary Information- Figure S4

Supplementary Information- Figure S5

Supplementary Information- Figure S6

## Data Availability

All immunoblot data generated in this study are provided in Supplementary Data. Other datasets generated and/or analysed during the current study are available from the corresponding author on reasonable request.
