# Supplementary material for: A WEEV 6K viroporin activates MLKL-dependent necroptosis to drive inflammatory injury
Source: Cell Death Discov. 2026 Jul 2;12:380. doi: 10.1038/s41420-026-03229-1 (PMC13597507; doi:10.1038/s41420-026-03229-1)
Supplement: Supplementary file 1 [file 41420_2026_3229_MOESM1_ESM.docx]

**Supplementary Information:**

**Supplementary Figure S1.** **Purification of wild-type WEEV 6K and the 6K^F34A^ mutant.**

**(A)** Purification of WEEV 6K proteins by Ni-NTA affinity chromatography. 10% SDS-PAGE gel stained with Coomassie blue (left) and Immunoblot analysis (right).

**(B)** WEEV 6K^F34A^ protein was purified using Ni-NTA affinity chromatography and resolved on a 10% SDS-PAGE gel followed by Coomassie blue staining. The red box highlights the bands corresponding to the target protein.

**Supplementary Figure S2.** **6K-induced cell death strictly depends on its channel conductance and intracellular calcium mobilization.**

**(A-B)** Effects of 6K and its mutants on HT22 cell viability. HT22 cells were transfected with an empty vector (Vector), wild-type 6K (6K), or various 6K point mutants (6K^F18A^, 6K^W20A^, 6K^C24A^, 6K^F34A^, 6K^R35A^). Cell survival (%) was evaluated by (A) CCK-8 assay and (B) ATP assay post-transfection. Data are presented as mean ± SEM; n = 4; **p* < 0.05, ***p* < 0.01, one-way ANOVA.

**(C)** Immunoblot analysis (left) and densitometric quantification (right) of p-RIPK1, RIPK1, p-RIPK3, RIPK3, MLKL and p-MLKL in HT22 cells transfected with empty vector (Vector), wild-type 6K (6K), or the 6K^F34A^ mutant. GAPDH was used as a loading control. Data are presented as mean ± SEM; n = 3; **p* < 0.05, Student’s *t-*test.

**(D-E)** Intracellular ATP levels in HT22 (D) and HEK293 (E) cells were measured 24 hours post-transfection. Cells were treated with BAPTA-AM (1 μM) at 6 hours post-transfection with 6K. Data are presented as mean ± SEM; n =4; **p* < 0.05, ***p* < 0.01, one-way ANOVA.

**Supplementary Figure S3. The IP₃R inhibitor 2-APB fails to reverse 6K-induced disruption of intracellular Ca²⁺ homeostasis, necroptosis activation, and cell death.**

**(A-B)** FDSS (fluorometric drug screening system) analysis of intracellular Ca²⁺ release from intracellular stores in HT22 (A) and HEK293 (B) cells 24 h after 6K transfection. Cells were treated with 2-APB (10 μM) at 6 hours post-transfection. Thapsigargin (TG; final concentration 10 μM) was added at 30 s (arrows) to trigger Ca²⁺ release. Quantification of Ca²⁺ responses shown in panels A and B (right). Data represent mean ± SEM, n = 4; **p* < 0.05, ***p* < 0.01, one-way ANOVA.

**(C-D)** Intracellular ATP levels in HT22 (C) and HEK293 (D) cells were measured 24 hours post-transfection. Cells were treated with 2-APB (10 μM) at 6 hours post-transfection with 6K. Data are presented as mean ± SEM; n = 4; **p* < 0.05, ***p* < 0.01, one-way ANOVA.

**(E)** Immunoblot analysis (left) and densitometric quantification (right) of p-RIPK1, RIPK1, p-RIPK3, RIPK3, MLKL and p-MLKL in HT22 cells transfected with 6K. Cells were treated with 2-APB (10 μM) 6 hours post-transfection, and protein levels were analyzed 24 hours after transfection. GAPDH was used as a loading control. Data are presented as mean ± SEM; n = 3; Student’s *t*-test.

**Supplementary Figure S4.** Representative fluorescence images showing HT22 cells transduced with control (Vector) or MLKL-targeting shRNA lentivirus, in the presence or absence of 6K expression. GFP fluorescence indicates successful viral transduction. Scale bar, 200 µm.

**Supplementary Figure S5. Transfection stress and 6K expression induce the accumulation of phosphorylated RIPK1 and RIPK3.** HT22 cells were left untreated (Blank), or transfected with an empty vector or a 6K-HA plasmid for 24 h. Protein levels of p-RIPK1, p-RIPK3, and HA were analyzed by immunoblotting, with GAPDH serving as a loading control.

**Supplementary Figure S6. Sequence alignment of human and mouse MLKL with WEEV 6K.**

**(A)** Full-length amino acid sequence alignment. Alignment of human MLKL (hMLKL), mouse MLKL (mMLKL), and Western equine encephalitis virus (WEEV) 6K protein. Numbers above the sequences denote amino acid positions. Identical and highly conserved residues are highlighted with red backgrounds and red text with blue frames, respectively. Dots (·) indicate gaps introduced for optimal alignment.

**(B)** Local sequence alignment of the MLKL N-terminal domain with the 6K transmembrane domain. Alignment between the truncated MLKL sequence containing the N-terminal domain and the WEEV 6K transmembrane domain. The highlighting scheme for conserved residues is the same as in (A).

**(C)** Monomeric and tetrameric structures of human MLKL. The left dashed box displays the conformation of the MLKL monomer, while the right dashed box illustrates the predicted tetrameric assembly. The N-terminal domain (residues 1-176) and C-terminal domain of MLKL are colored pink and cyan, respectively. Structural comparison between the AlphaFold-predicted models of human MLKL and WEEV 6K revealed limited similarity, with an RMSD of 4.717 Å following alignment in PyMOL.

**(D)** Structural superimposition of human MLKL (hMLKL) and WEEV 6K. Predicted structures of the hMLKL tetramer (pink) and WEEV 6K hexamer (green helices) are aligned, showing their relative spatial positioning. All 3D protein models were generated using AlphaFold.
